# The Irish Lunacy Blue Book

**Published:** 1894-04-07

**Authors:** 


### IRISH LUNACY BLUE BOOK.

The forty-second report of the inspectors of lunatics for Ireland is addressed to Lord Houghton in his capacity of Lord Lieutenant, and his Excellency is informed that the Island contained on January 1st, 1893, a total of 17,124 lunatics, showing an increase during the year of 436. Of the total 16,331 are paupers, 644 are private patients, and 149 are criminal lunatics. These numbers do not include lunatics wandering at large, nor lunatics in private dwellings. The latter cannot be considerable, but the census of 1891 showed that the former reached the respectable figure of 4,970. One of the most deplorable things revealed in these Blue Books, and the monotonous tale is told yearly in every division of the kingdom, is the increase in the number of the registered insane, and the sad fact has to be chronicled that, so far as can be gathered from these reports, nothing is being done in the way of prevention of the disease. And yet this very Blue Book tells us that drink and hereditary influence caused the insanity in 1,020 of the 3,181 admissions of the year. Someone lately proposed that the marriage ceremony should be in the hands of medical men and not with the clergy. How this would work as a general rule we do not know, but it is certain that in another generation hereditary insanity would be unknown, and a good many other diseases would be relegated to the limbo of forgotten text books.

In England with a rapidly increasing population, proportionate increase of insanity is to be looked for, but it is the same in Ireland with a decreasing population. In 1881 there were 9,774 lunatics, and in 1891 the number had risen to 14,945; and if we go back to 1851 the figures are more instructive still. In that year there was one lunatic to every 657 of the population; in 1861 the ratio had risen to 1 in 411; in 1871 it was 1 in 328; in 1881 it was 1 in 281; and in 1891 it had got as high as 1 in 222! In the county of Meath the ratio was 1 in 126. This is probably the highest ever reached in any country. In County Down it was 1 in 333. The latter approaches very closely to the English ratio, namely, 30·21 in 10,000 of the population. It would seem the Irish Celt is about twice as liable to insanity as the English Saxon.

The Commissioners adhere to their opinion that the increase of insanity in Ireland is both absolute and relative, and on examination of the reason we admit they have a strong case if not an unanswerable one.

In some respects the Lunacy Law of Ireland differs from that of England and Scotland. In England, for instance, it is the duty of the relieving officer to find out how far the lunatic or his friends can contribute to his support in the asylum; but in Ireland this duty is thrown on the asylum officials, and must, therefore, be almost a dead letter. Then a large proportion of the insane are sent to district asylums under the Dangerous Lunatics Act, and are practically prisoners for whom no charge at all can be made. Further, transfers cannot be made from one asylum to another in Ireland, and it must often happen that lunatics are supported by a district having nothing to do with them, while their own counties are let off scot free.

The percentage of recoveries to admissions stands at 37·2; England being 38·94, and Scotland 42·66.

The death rate in Ireland is 8·3 on the average number resident. In England it is 9·98, and in Scotland 8·3. So that in the low recovery rate and low death rate we have an explanation of part of the increment of Irish lunatics. The total number of deaths was 995, and post-mortem examinations were made in 198 instances, a number which though small in comparison with English asylums is higher than in any former year, and the Commissioners think these examinations are of "much importance for the protection of the insane, and for the furtherance of the scientific study of insanity."

There were only four suicides during the year in Irish asylums, being at the proportion of 2·33 per ten thousand. This is by no means large, but it is higher than that of English county and borough asylums, which stands at 1·94 per ten thousand, while Scotland had something like a ratio of 6·89 per ten thousand.

The Commissioners say that the accommodation in the district asylums is quite inadequate to supply the wants of the insane population, and certainly their figures show an amount of overcrowding that would hardly be tolerated in England. One of the worst asylums in this respect is that of Richmond, near Dublin. It contains 1,500 patients, and there is room for 1,100 only. In 1891 a step in the right direction was made when it was decided to abolish visiting physicians and substitute assistant medical officers.

The Commissioners still lament the absence of hospitals for the insane on the lines of our English lunatic hospitals, but they would be conferring a much greater boon on the middle-class people by endeavouring to obtain a law compelling district asylums to build for a certain number of private patients. By this step the deserving classes would really get the room they require, and the profits would help to reduce the burden on the ratepayer in his support of the pauper lunatic.

The average cost per head per annum is in Irish asylums £23 0s. 8d., in Scotch asylums £23 8s. 6d., and in English asylums £23 16s. 8d.

